# In Vitro Evaluation of Antibacterial Properties and Smear Layer Removal/Sealer Penetration of a Novel Silver-Citrate Root Canal Irrigant

**DOI:** 10.3390/ma13010194

**Published:** 2020-01-02

**Authors:** Riccardo Tonini, Massimo Giovarruscio, Fabio Gorni, Andrei Ionescu, Eugenio Brambilla, Irina Makeeva Mikhailovna, Arlinda Luzi, Paula Maciel Pires, Salvatore Sauro

**Affiliations:** 1 Department of Endodontics and Restorative Dentistry, School of Dentistry, University of Brescia, 25125 Brescia, Italy; riccardotonini@me.com; 2 Department of Therapeutic Dentistry, Sechenov University of Moscow, 119435 Moscow, Russia; giovarruscio@me.com (M.G.); irina_makeeva@inbox.ru (I.M.M.); 3 Division of Peri-Oral Aesthetics, School of Dentistry, University Vita Salute San Raffaele, 20132 Milano, Italy; fabiogorni@dentalservicesanzio.it; 4 Oral Microbiology and Biomaterials Laboratory, Department of Biomedical, Surgical, and Dental Sciences, Università degli Studi di Milano, 20133 Milan, Italy; AndreiIonescu_40@hotmail.com (A.I.); eugenio.brambilla@unimi.it (E.B.); 5 Departamento de Odontología, Facultad de Ciencias de la Salud, Universidad CEU-Cardenal Herrera C/Del Pozo s/n, Alfara del Patriarca 46115 (Valencia), Spain; arlinda.luzi@uchceu.es (A.L.); paulinha_pmp@hotmail.com (P.M.P.); 6 Pediatric Dentistry, Universidade Federal do Rio de Janeiro, 21941-901 Rio de Janeiro, Brazil

**Keywords:** antibacterials, root canal dentin, smear layer, enterococcus faecalis, scanning electron microscopy, confocal macroscopy, silver ions, citric acid

## Abstract

This study aimed at evaluating the efficacy of a novel silver-citrate root canal irrigation solution (BioAKT) on smear layer removal, sealer penetration after root canal instrumentation and antibacterial activity. Single-root teeth were endodontically treated, sealed with an epoxi-amine resin sealer and irrigated using: Group I: 5.25% sodium hypochlorite (NaOCl); Group II: silver-citrate solution (BioAKT); Group III: phosphate buffer solution (PBS); Group IV: 17% ethylenediaminetetraacetic acid (EDTA). Smear layer removal and silver deposition at the coronal, middle and apical portion of each canal were analyzed using scanning electron microscopy (SEM) and energy-dispersive x-ray spectroscopy (EDS). Sealer penetration into dentinal tubules at coronal, middle and apical portion was assessed through dye-assisted confocal microscopy (CSM). Both SEM and CSM micrographs were evaluated by two examiners (κ = 0.86), who were blind to the irrigation regimens; scores were given according to the degree of penetration of the sealer. Data analysis included Pearson’s *x*^2^ and Sidak’s multiple comparisons. Dentin discs were polished and sterilized. *Enterococcus faecalis* biofilms were grown using a continuous-flow bioreactor under anaerobic conditions for 72 h. Specimens were irrigated with the tested solutions, and bacterial viability was assessed using a tetrazolium salt assay (MTT). Statistical analysis included one-way ANOVA and Student’s post-hoc t-test (p < 0.05). BioAKT and EDTA were the most efficient solutions both in removing the smear layer and allowing sealer penetration. However, at the apical portion BioAKT performed significantly better compared to EDTA both in smear layer removal and sealer penetration (p < 0.05). BioAKT and NaOCl showed comparable antibacterial effect (p = 0.53). In conclusion, BioAKT represents a suitable smear layer removal agent, which allows for reliable sealer penetration at the apical portion of the root canal system and offers significant antibacterial properties.

## 1. Introduction

One of the most challenging tasks in endodontic treatments is the accomplishment of a comprehensive elimination of indwelling microorganisms, as well as a suitable sealing of the root canal system [1]. The fact that mechanical endodontic instrumentation allows clinicians to obtain a bacteria-free root canal system by itself has no evidence-based support in modern literature [2]. Indeed, it is well documented that most clinicians rely on a successful preparation of the entire length of the root canals through appropriate mechanical instrumentation, along with the use of appropriate irrigation protocols able to remove organic/inorganic smear layer and indwelling microorganisms [3]. However, if one considers the anatomical complexity of root canals (e.g., lateral/accessory canals, apical ramifications and severe curvatures), comprehensive decontamination of the root canal system would be quite a challenge, unless specific irrigation/disinfection protocols are properly employed [4]. Furthermore, it must be considered the essential role of a proper sealing of endodontically-treated root canals using cements and other specific restorative materials in preventing marginal leakage and re-infection of root canals [5,6].

Sodium hypochlorite (NaOCl) is widely used as an endodontic irrigation agent due to its antibacterial and proteolytic action, which allows clinicians to dissolve necrotic pulpal tissue within the root canal system [7]. Moreover, its use is often associated with a calcium chelating agent such as ethylenediaminetetraacetic acid (EDTA) due to the ineffectiveness of NaOCl to remove inorganic smear layer [8]. Although NaOCl is the main proteolytic irrigation agent in endodontics, it must be used with extreme caution as it is highly cytotoxic [9] and presents potential side effects and safety concerns. Indeed, its improper use may cause serious clinical complications (e.g., oedema, ecchymosis, tissue necrosis and paresthesia) [10] if extruded throughout the apical foramen [11]. Another disadvantage of NaOCl is its ability to induce peritubular and intertubular erosion by proteolytic degradation, especially when used in combination with EDTA [12]. Indeed, it has been shown that NaOCl induces severe structural changes of the dentinal collagen; the formation of fragile, spongy-like root dentin is commonly observed during ultramorphology studies [13]. Such a situation may jeopardize the longevity of the root canal treatment, especially where the remaining tooth structure is drastically reduced during endodontic instrumentation and preparation of the cavity and the access to canals [14]. Furthermore, when used in combination with NaOCl, EDTA reduces the availability of the hypochlorite anion in solution, making NaOCl less effective in removing the necrotic pulp tissue, as well as reducing its antibacterial activity [15]. In view of all these facts, it is clear that innovative irrigation agents are required in endodontics.

A myriad of compounds dissolved in aqueous solutions, including saline or organic acids (e.g., citric, lactic, tannic and polyacrylic acid), or chelating solutions like bis-dequalinium acetate, natural polysaccharide (0.2% chitosan) and broad-spectrum antibiotics such as tetracyclines [16], have been suggested as alternative root canal irrigants able to remove the smear layer and kill as many microbial species as possible. In particular, anaerobic gram^-^ bacteria such as *Enterococcus faecalis* have frequently been isolated from pathological root canals. This microorganism is currently considered one of the most resistant species responsible for failure of root canal treatments [9]. Recently, silver-based active principles dispersed in aqueous solutions have captured the attention of researchers and clinicians due to their powerful biocidal properties upon contact on many microorganisms [17,18] that could be ideal for an innovative root canal irrigant.

The aim of this in vitro study was to evaluate the efficacy of an innovative silver-citrate endodontic metabolic substrate on smear layer removal and sealer penetration at different root canal portions (e.g., apical, middle, cervical) after endodontic instrumentation compared to 17% EDTA and 5.25% NaOCl. Moreover, the antibacterial activity of such an innovative silver-citrate endodontic irrigation agent was also evaluated against a mature (72 h) *E. faecalis* biofilm and compared to that of NaOCl.

The first tested hypothesis was that such a novel irrigation agent would be able to remove more efficiently the smear layer and achieve better sealer penetration at all root canal depths compared to EDTA and NaOCl. The second hypothesis was that such a novel silver-citrate endodontic metabolic substrate would have comparable antibacterial activity against *E. faecalis* biofilm to 5.25% NaOCl.

## 2. Materials and Methods

### 2.1. Evaluation of Smear Layer Removal though Scanning Electron Microsopy (SEM) 

An institutional ethical committee approval (IEC: CEI17/018) was obtained to use extracted human teeth for research purposes. In order to standardize our study, only single-root teeth (upper canines and incisors) with intact crowns and roots, extracted for periodontal reasons were collected and used in this study under an informed consent approved by every single donor. Teeth with curved roots or endodontically treated were excluded from the study. After extraction, teeth were cleaned by removing the remaining soft tissue using a manual scaler and stored in distilled water solution at 4 °C no longer than three months.

Endodontic access was made on each tooth using a dental turbine equipped with a diamond bur. A size 10 K-file was placed into the root canal until the tip was just visible at the apical foramen, then the working length was set at 1 mm from the apex. Further instrumentation was performed according to the conventional step-back preparation using endodontics files from size #15 to #35. During instrumentation, the canals were only irrigated with 3 mL phosphate buffer solution between every instrument, using 29-gauge needles (Ultradent Prod Inc., South Jordan, UT, USA) that were kept 2 mm away from the working length.

After root canal instrumentation, the root specimens were randomly divided into four groups (n = 10 teeth/group) based on the irrigation solution used in this study. Group I: 5.25% NaOCl (Ogna Laboratori Farmaceutici, Muggiò, Italy); Group II: Silver citrate dihydrate (silver < 100 ppm; citric acid < 1000 ppm) in water solution (BioAKT metabolic substrate, New Tech Solutions s.r.l., Brescia, Italy); Group III: 17% EDTA (PULPDENT, Watertown, MA, USA); Group IV: phosphate buffer solution (PBS). All specimens received a standardized protocol for irrigation only at the end of the root canal instrumentation (10 mL for 1 min) and finally dried with paper points [19].

Subsequently, the roots were split in two parts. Guide grooves were made longitudinal along the mesial and distal surfaces of the specimens with a double-sided diamond discs, and finally, they were split in two parts using some cutting pliers to expose root canals. Subsequently, the hemisection specimens were mounted on a metallic stub using conductive glue, desiccated overnight in a vacuum-pump cell, gold-sputtered, and finally examined at the coronal, middle and apical portion of the root using scanning electron microscope (SEM: FE-SEM S-4100; Hitachi, Wokingham, UK) in secondary electrons mode at 10 kV and a working distance of 15 mm. Energy dispersive x-ray spectroscopy (EDS, INCA Energy, Oxford Instruments, High Wycombe, UK) was used for chemical microanalysis to detect the presence of silver deposits on the root dentin after irrigation using the four different solutions tested in this study. Five representative digital photomicrographs from each root portion were obtained in each root fragment. These images were evaluated by two examiners who were blind to the irrigation protocols and scores were given according to a modified version of the criteria suggested by Rome et al. [20], Table 1. Each image was considered as a statistical unit, since a single value was assigned to each of them. The level of agreement among examiners was assessed by the Kappa test, which presented an overall result of (κ = 0.86). Differences between the different irrigants were determined using Pearson *X*^2^ (chi-square) test and Sidak’s test for multiple comparison, with significance predetermined at α = 0.05.

### 2.2. Sealer Penetration: Confocal Microscopy (CSM) Assessment

Further single-root teeth (n = 10 teeth/group) were instrumented and irrigated as previously described. Subsequently, #40 master gutta-percha cones were applied in combination with 10 microliters of root canal sealer (AH Plus, Dentsply-DeTrey, Konstanz, Germany), labeled with 0.1% Rhodamine B dye (Sigma-Aldrich, St. Louis, MO). The latter was applied using a #20 Lentulo spiral [21]; master cones once positioned were adapted to the root canals using the lateral condensation technique. The specimens were then submitted to radiographic examination in both mesiodistal and buccolingual directions, to check the quality of the fillings. All the specimens were stored at 37 °C and 100% humidity for 7 days to allow the sealer to set completely. They were subsequently embedded in epoxy resin and cross-sectioned to obtain discs (1.5 mm thickness), starting 3 mm from the apex, using a diamond-embedded saw (Buehler Ltd., Lake Bluff, IL, USA) coupled to an automatic microtome (Remet evolution, REMET, Bologna, Italy) under continuous water irrigation. Afterwards, the specimens were ultrasonicated in distilled water for 5 min and then polished for 30 s each side with a 2400-grit SiC paper. The specimens were finally ultrasonicated again in distilled water for 3 min and submitted for CSM analysis. Using a confocal scanning microscope (Olympus FV1000, Olympus Corp., Tokyo, Japan) equipped with a Plan-Apochromat 10×/0.45 NA objective lens and using a 543 nm LED illumination. Reflection and fluorescence images were obtained with a 1-μm z-step to optically section the specimens from 5 μm to a depth of up to 20 μm below the surface. The z-axis scan of the interface surface was pseudo-colored arbitrarily for improved exposure and compiled into both single and topographic projections using the image-processing software (Fluoview Viewer, Olympus Corp., Tokyo, Japan). The configuration of the system was standardized and used at constant settings for the entire investigation [22]. Each dentin interface was investigated completely, and then five z-stack images were randomly captured. Single-projection reconstructions from each z-stack representing the most common morphological features observed along the bonded interfaces were obtained. These images were evaluated individually by two examiners who were blind to the irrigation regimens and scores were given according to the criteria provided in Table 2. The level of agreement among examiners was described above in Section 2.1. Differences between the groups were determined using Pearson *X*^2^ test and Sidak’s test for multiple comparison, with significance predetermined at α = 0.05.

### 2.3. Biofilm Formation and Microbiological Assessment

Thirty-two extracted molars were used to prepare one dentin crown disc from each tooth (6.0 mm diameter) using a diamond-embedded saw (Buehler Ltd.), coupled to an automatic microtome (REMET) under copious water irrigation. Dentin crowns specimens were used instead of root canal dentin in order to uniformize biofilm development over flat specimens’ surfaces and to standardize the contact of the irrigant solution with the dentin surface. Such specimens were oriented with the inner dentin exposed on the upper side and polished with 600 and 1200-grit SiC papers; these were finally cleaned for 5 min in an ultrasound bath containing distilled water. 

A strain of *Enterococcus faecalis* ATCC 29212 was cultured overnight in brain-heart infusion broth (BHI) at 37 °C in anaerobic conditions, giving a final cells concentration of 1.5 × 10^8^ colony forming units (CFU)/mL. A biofilm was obtained on the dentin surfaces using a continuous flow bioreactor under anaerobic conditions for 72 h. In brief, for simulation of biofilm formation, a modification of a commercially available Drip Flow Reactor (DFR 110; Bio-Surface Technologies, Bozeman, MT, USA) was employed as continuous flow bioreactor. The modified design allowed the placement of customized specimen trays on the bottom of the flow cells and the complete immersion of the surfaces of the specimens into the surrounding flowing medium [23]. Dentin crowns specimens were randomly distributed across two Teflon trays, which fixed the specimens tightly and exposed their inner dentin surfaces to the surrounding medium; the trays were press-fitted on the bottom of each flow cell of the reactor. Prior to the experiments, all tubing and specimen-containing trays together with the dentin crowns specimens were sterilized using a chemiclave with hydrogen peroxide gas plasma technology (Sterrad; ASP, Irvine, CA, USA). Heat-related damage of the specimens was avoided by limiting the maximum temperature to 45 °C. The whole reactor was assembled inside a sterile hood and transferred into an incubator to operate under a standardized temperature of 37 °C. A total of 10 mL of *Enterococcus faecalis* (overnight culture) was inoculated in each flow-cell. After a 4 h period to allow bacterial adherence and initial colonization of the surfaces, a computer-controlled multichannel peristaltic pump (RP-1; Rainin, Emeryville, CA, USA) was turned on to provide a continuous flow of nutrients through the flow-cells, at a speed of 9.6 mL/h, for 72 h. A simulated pulpal fluid (SPF), containing 30 mmol/L Hepes buffer, 0.93 mmol/L CaO, 0.60 mmol MgO, 77.6 mmol/L, 1.1 mmol/L H_3_PO_4_ and 20% albumin was used as a nutrient source [24].

After 72 h, specimens were removed from the bioreactor, washed three times with sterile PBS, and randomly assigned (n = 8/group) to one of the four treatment groups, then washed with 10 mL of the respective irrigation solution for 1 min. After that, specimens were gently rinsed with sterile PBS for 5 min; bacterial viability was assessed using a tetrazolium salt (MTT)-based viable biomass assay.

### 2.4. Viable Biomass Assay

The assay was conducted as previously described [25]. Briefly, two stock solutions were prepared by dissolving 5 mg/mL of 3-(4,5)-dimethylthiazol-2-yl-2,5-diphenyltetrazolium bromide (MTT) in sterile PBS, and 0.3 mg/mL of N-methylphenazinium methyl sulphate (PMS) in sterile PBS. All tested solutions were stored at 2 °C in light-proof vials until the day of the experiment when a fresh measurement solution (FMS) was made by mixing 1 mL of MTT stock solution, 1 mL of PMS stock solution and 8 mL of sterile PBS. A lysing solution (LS) was prepared by dissolving 10% v/v of sodium dodecyl sulphate and 50% v/v of dimethylformamide in distilled water. The treated and rinsed discs were placed on the bottom of 48-well flat-bottom plates. Subsequently, a total of 300 µL of FMS were pipetted into each well, and the plates were incubated at 37 °C in light-proof conditions for 3 h. During incubation, electron transport across the microbial plasma membrane and, to a lesser extent, microbial redox systems converted the yellow MTT salt to insoluble purple formazan; the conversion was facilitated by the intermediate electron acceptor (PMS) [26]. The unreacted FMS was gently removed from the wells by aspiration, and the formazan crystals were then dissolved by adding 300 µL of LS into each well and further incubating at room temperature in lightproof conditions for 1 h. A total of 100 µL of the suspension were then removed from each well, and optical density (550 nm) was measured with a spectrophotometer (Genesys 10-S, Thermo Spectronic, Rochester, NY, USA). Data were submitted to statistical analysis, including verification of normality of distribution and homoscedasticity; then, a one-way ANOVA and a Student’s post-hoc t-test were performed, setting the significance at α = 0.05.

## 3. Results

**Figure 1:**
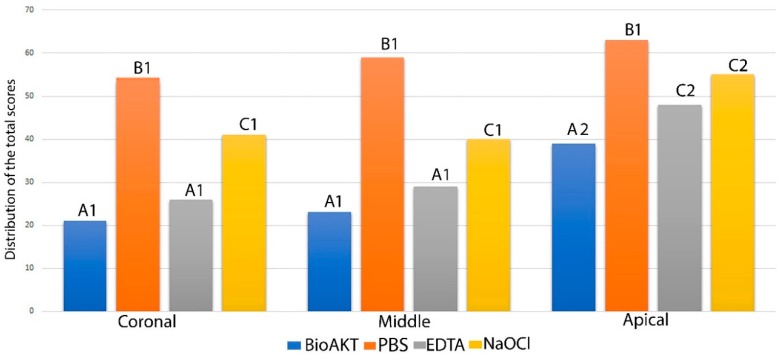
Graph showing the quantitative total distribution of the scores for smear layer removal (score 0–4) obtained during the SEM evaluation for each treatment at different root canal level. Please note that the lower the total value the greater the ability to remove smear layer. Similar letter indicates no significance between the irrigants in each third level of the root. Similar number indicates no significance between the same irrigant in different third level.

**Figure 2:**
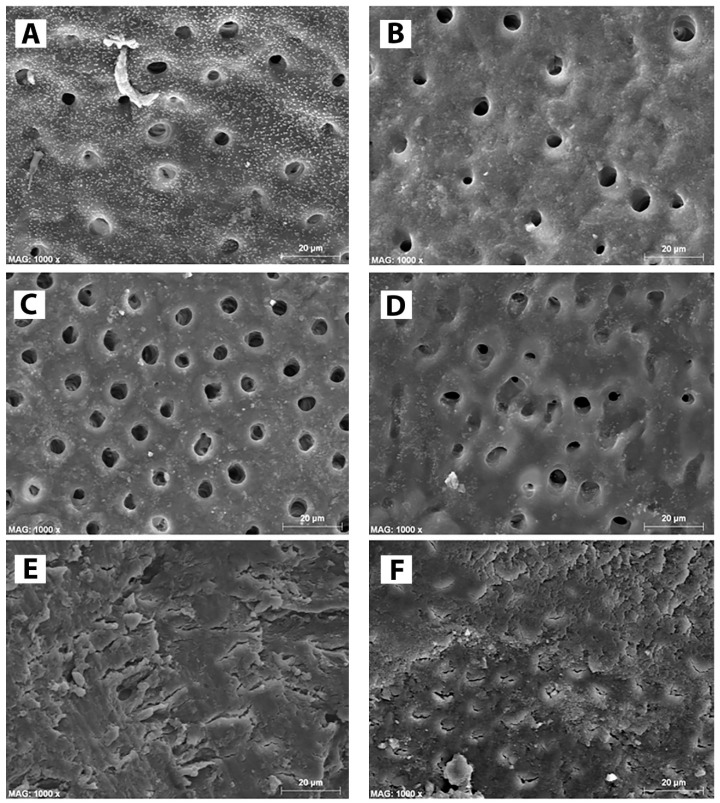
SEM micrographs of root canal dentin treated with the irrigation solutions tested in this study. (**A**) note the effect of BioAKT on the coronal part of the root canal dentin where the smear layer was totally removed and most of the dentinal tubules exposed. (**B**) The same situation was observed on the middle part of the root canal after treatment with BIOAKT. EDTA was able to remove the smear layer and expose dentinal tubules both at coronal dentin (**C**) and middle dentin (**D**). (**E**) The control irrigant (PBS) had no removal effect on any part of the root canal dentin; note the presence of a compact smear layer with no dentinal tubules exposed. (**F**) Representative image of coronal and middle dentin treated with NaOCl showing shallow removal effect on the smear layer. Note that most of the root surface remined covered by a porous organic smear layer with very few dentinal tubules, which were only partially exposed.

**Figure 3:**
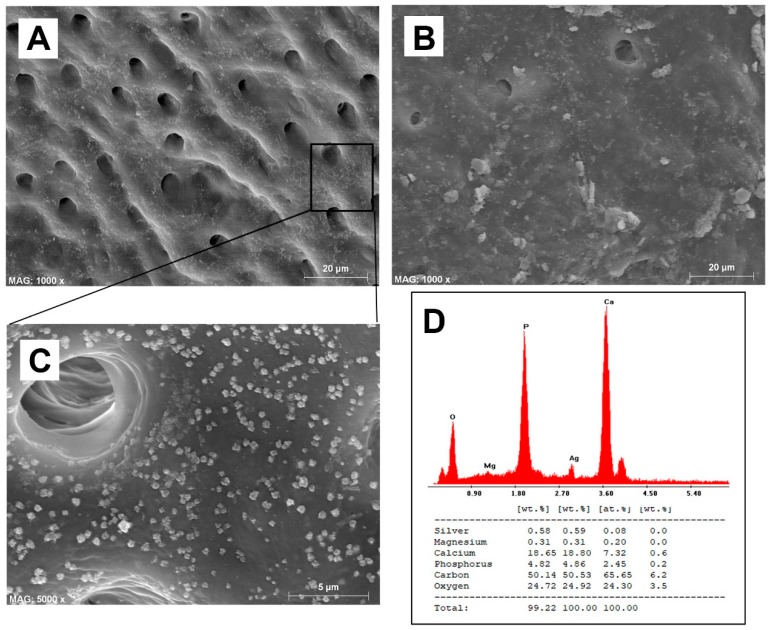
The effect of BioAKT on the apical part of the root canal dentin; the smear layer was well removed and most of the dentinal tubules exposed. (**B**) Note the effect of EDTA on the apical part of the root canal dentin where the smear layer was only partially removed, leaving most of the dentinal tubules still obliterated. (**C**) Higher magnification of image (**A**) where possible to observe the presence of nanometric mineral precipitations on dentin surface. (**D**) EDS analysis confirmed that the mineral clusters observed in image (**C**) contained calcium, phosphorous, magnesium and silver.

The quantitative results for smear layer removal obtained during the SEM evaluation are depicted in Figure 1, while the SEM ultramorphology results are shown in Figure 2A–F. It was observed that BioAKT and EDTA presented no significant difference in removing the smear layer at the coronal and middle portions of the root canal (p > 0.05), while NaOCl had greater ability in removing the smear layer only compared to the control group (PBS) (p < 0.05). On the other hand, at the apical portion of the root, BioAKT showed the highest ability to remove the smear layer and expose dentinal tubules (Figure 3A), when compared to EDTA (Figure 3B, p < 0.05); there was no significant difference between EDTA and NaOCl (p < 0.05). The results showed that each irrigation solution tested in this study, excluding the control group PBS, showed a significantly lower ability in removing the smear layer at the apical root canal level compared to the middle and cervical thirds (p < 0.05). It was interesting to notice, during SEM-EDS assessment, the presence of nano-agglomerations on the root dentin treated with the bioAKT (Figure 3C), which were principally constituted of silver, calcium, phosphorous and magnesium (Figure 3D); this was not observed in any specimen of the other groups.

**Figure 4:**
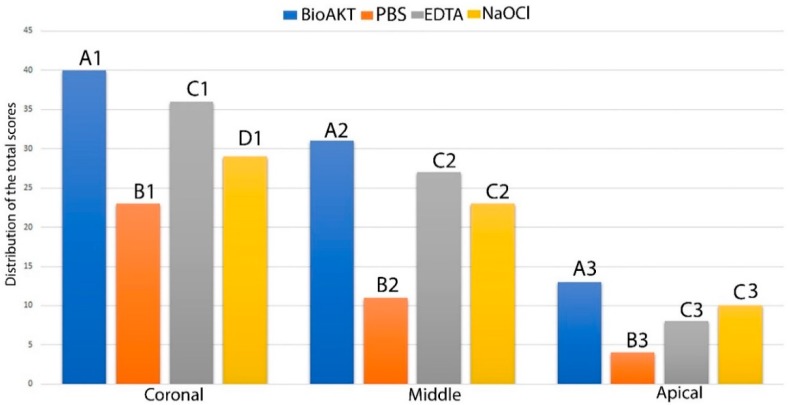
Graph showing the distribution of the scores for sealer penetration (score 0–3) obtained during the confocal microscopy assessment for each treatment at different root canal level; the higher the total value the greater the ability to remove smear layer. Similar letter indicates no significance between the irrigants in each third level of the root. Similar number indicates no significance between the same irrigant in a different third level.

**Figure 5:**
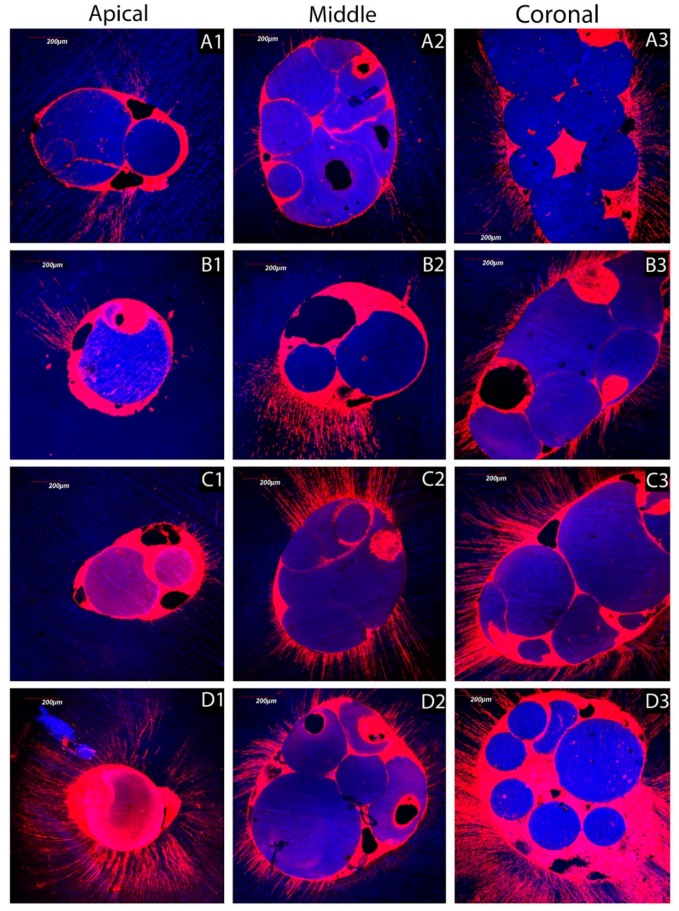
Confocal z-stack images converted into single-projection images, which show sealer penetration at different levels of the root canal dentin treated with the irrigation solutions tested in this study. (**A1**) (apex), (**A2**) (middle) and (**A3**) (coronal) show the poor penetration of the sealer into the root canal dentin treated using the control PBS. (**B1**) (apex) and (**B2**) (middle) show an evident lack of penetration of the sealer in most of the root canal dentin treated using NaOCl, but with some more sealer penetration at the coronal level (**B3**). (**C1**) (apex) shows an evident lack of penetration of the sealer in particular at the apical level of the root canal dentin treated using EDTA. However, a decent sealer penetration is evident at middle (**C2**) and coronal (**C3**) dentin. (**D1**) (apex), (**D2**) (middle) and (**D3**) (coronal) show a clear penetration of the sealer in root canal dentin treated using the BioAKT silver citrate irrigation agent.

The results of sealer penetration obtained during confocal microscopy assessment are shown in Figure 4, while the confocal images can be observed in Figure 5. It was interesting to observe, in all groups, a different penetration of the sealer at all root canal regions (e.g., coronal, middle and apical); in other words, all the irrigants used in this study performed much better at coronal > middle > apical part (p < 0.05, Figure 5). However, at all root dentin levels, the novel silver-citrate irrigation solution showed the highest (p < 0.05) ability in allowing a more uniform sealer penetration (Figure 5D1–D3). Conversely, the control PBS group showed the lowest efficacy (p < 0.05) in terms of sealer penetration. The comparison between EDTA and NaOCl resulted in no significant difference (p > 0.05) at any root dentin levels (p > 0.05).

**Figure 6:**
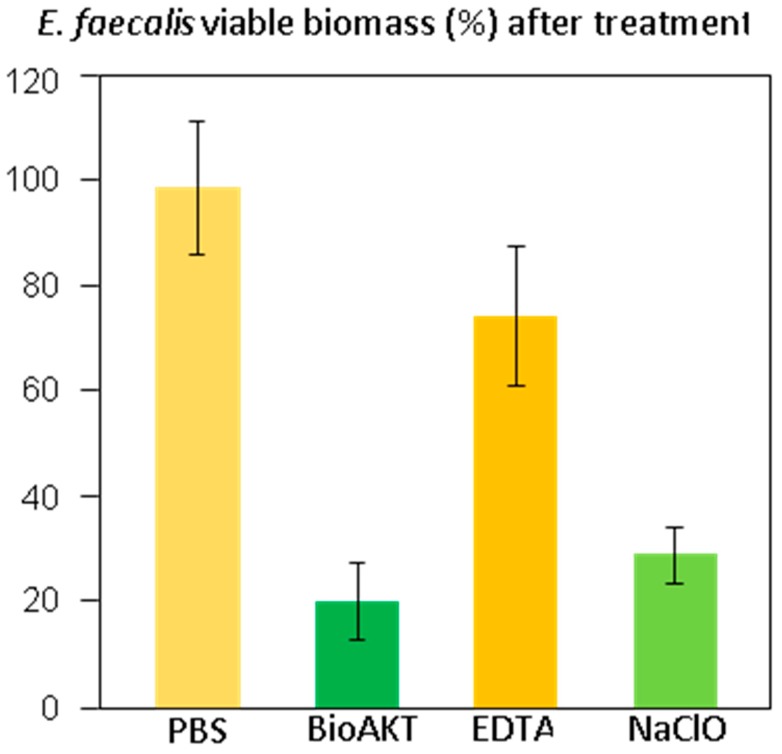
This graph shows the viable *Enterococcus faecalis* biomass adherent to the dentin surfaces after the different treatments. Results are given in % of viable biomass ± 1 standard deviation, assuming PBS treatment as 100% viability and after removing the blank.

The results of the viable biomass assay are displayed in Figure 6. Shapiro-Wilk’s test was used to assess normality of distribution (p = 0.0583). Levène’s test was used to assess homoscedasticity (p = 0.3598), which confirmed the homogeneity of variances among groups. ANOVA showed the existence of highly significant differences between groups (p < 0.0001). Student’s t-test demonstrated that specimens treated with the novel metabolic substrate BioAKT, and NaOCl presented a significantly lower amount of viable biomass compared to the other groups (p < 0.0001). No significant difference was found between the efficacy of BioAKT and NaOCl (p = 0.54), suggesting that both solutions had good efficacy against *Enterococcus faecalis* biofilm. However, none of the tested solutions achieved complete eradication of the biofilm after 1 min irrigation, confirming the robustness of the biofilm generated by the tested bacterial strain.

## 4. Discussion

It is crucial to consider that most of the irrigation solutions used during root canal treatments modify the chemo-mechanical properties of the root canal dentin, thus affecting the performance and longevity of all materials used for root canal obturations and restorations [27,28,29]. Nevertheless, NaOCl remains a “unique” endodontic irrigant due to its ability to offer excellent lubrication and dissolution of organic tissue (e.g., pulpal tissue and bacteria), as well as to its wide-ranging spectrum of activity against microorganisms. It has been advised that the higher the concentration of NaOCl, the better the antibacterial and tissue dissolution effects during root canal treatment. This is the reason why a 5.25% NaOCl solution was used in this study [1]. The results of this study showed that even at the tested concentration, NaOCl was able to remove only partially the smear layer from the root dentin surface (Figure 2F), although at the coronal and middle portions of the root- it was possible to observe root canal sealer penetration into the dentin tubules at some extent (Figure 5B2,B3). Conversely, at the apical portion of the root canal, such an irrigant was not effective in removing the smear layer and in favoring sealer penetration into the dentin tubules (Figure 5B1). In contrast, the microbiological evaluation confirmed the highly effective antibacterial activity of NaOCl on a biofilm of *Enterococcus faecalis* (Figure 6). Our results agree with those of previous studies showing that a 5.25% NaOCl solution was able to remove organic and loose superficial debris but was not totally effective in removing inorganic smear layer [30,31].

Citric acid is a weak organic acid with strong chelating properties, which is commonly used in periodontal therapy as dentin conditioner after scaling and root planning, or in restorative dentistry as etchant agent in some protocols before adhesive application. Indeed, the ability of citric acid to decalcify dental hard tissues is due to the chelation of Ca^2+^ ions at a slightly acidic pH environment [32]. Citric acid solutions have been also employed as endodontic irrigants with a concentration between 25% and 50%. However, recent studies demonstrated that citric acid solutions with a lower concentration (<10%) could offer comparable results to those obtained with 17% EDTA [33,34].

In the current study a novel metabolic substrate based on silver citrate (BioAKT) was tested as an innovative endodontic irrigation solution, (pH ~ 1.7). While citric acid possesses the aforementioned activities that make it an interesting active principle for an endodontic irrigant formulation, it can also be very effective as a capping agent for silver ions and nanoparticles, thus stabilizing the formulation [35]. Our SEM evaluation showed that the root canal dentin treated with BioAKTwas able to remove smear layer and expose most of the dentinal tubules in the coronal (Figure 2A) and middle portions of the root canal dentin (Figure 2B); the 17% EDTA solution tested in this study showed comparable results both at coronal (Figure 2C) and middle dentin (Figure 2D). However, despite a relatively low concentration of citric acid, BioAKT was the most efficient solution in removing the smear layer (Figure 3A), allowing for a greater sealer penetration at the apical area of the root (Figure 5D1). Our results are in partial agreement with those of Machado et al. [33], who reported that sealer penetration into the dentinal tubules increased in all root thirds when the specimens were treated with citric acid or EDTA, but the performance at the apical portion was similar, irrespective of the chelating solution employed. These controversial outcomes might be a consequence of the endodontic obturation approach performed by those authors, who used a lateral and vertical condensation technique to adapt the sealer along the surfaces of the root canal dentin, increasing its penetration within the dentinal tubules at the apical portion of the root canal specimens. Another difference in methodology that could explain these different outcomes may be associated to the different irrigation protocol used in our study, where standardized irrigation using the tested solutions was only performed at the end of the mechanical root canal instrumentation (10 mL for 1 min). Other studies showed no direct correlation between smear layer removal and root canal sealer penetration, as there was no significant difference between apical, middle and coronal portions of the tested specimens [36,37]. In our study, such differences may be due to the improved physico-chemical properties and flowability of the chosen root canal sealer [38].

One of the most relevant results obtained when using BioAKT was the presence of nanometric precipitations (Figure 3C) constituted principally by calcium, phosphorous, silver and magnesium (Figure 3D). We speculate that those silver-containing crystallites are the result of the activity and interaction rates of silver ions contained in the solution. Such high activity may explain its strong antibacterial effect against mature *E. faecalis* biofilm (72 h), which was comparable to that obtained using NaOCl (Figure 6). A possible elucidation to the mechanism of action of silver on an *E. faecalis* biofilm is that silver ions and nanoparticles may be able to interact and penetrate the cell membrane of both Gram-positive and Gram-negative bacteria, causing damage to deoxyribonucleic acid and finally death of bacteria by liberating silver ions. Moreover, silver may have an intrinsic peroxidase-like activity, which can catalyze hydrogen peroxides (PBS_2_) to generate free radicals in a pH-dependent manner [31,39]. Yamaguchi et al. [40] reported that low concentration (0.5, 1, 2 M) citric acid solutions could have an antimicrobial potential against anaerobic bacteria species when employed as potential irrigation solutions for root canals. The existence of a synergistic antimicrobial effect between citric acid and silver ions, allowing for a relatively high antimicrobial activity at relatively low concentrations of the active principles is an inspiring possibility that might be extensively addressed in future studies.

However, some disadvantages can be related to the use of silver ions and nanoparticles in endodontics; for instance, as a consequence of their prompt interaction with biological tissues, they can induce dentin discoloration and present cytotoxicity at some extent due to their size, composition and surface properties such as a high surface-to-volume ratio and non-specific oxidative injuries [41,42]. The toxicity of silver ions can become relevant in a concentration range of 1–10 mg/L, while silver nanoparticles are usually toxic for eukaryotic cells when used in solutions at 10–100 mg/L [43].

Our current study also showed that EDTA presented no evident antibacterial effect, although it was much more efficient than NaOCl in removing the smear layer and allowed sealer penetration in all root canal parts. EDTA has been generally used to affect and dissolve the mineral phase of dentin without altering its collagen part; a stable complex is formed with calcium ions until an equilibrium is reached, so that no further demineralisation/chelating reaction will take place [44]. However, previous studies [8] reported that a consecutive combination of EDTA and NaOCl may cause substantial erosion along the surfaces of the root canal dentin. Furthermore, the superficial erosive effect of NaOCl on root canal dentin surfaces is considered irreversible, irrespective of the association with EDTA as consecutive irrigants [45,46].

Scanning electron microscopy is one of the most appropriated methods to assess the ability of endodontic irrigation solutions in removing smear layer in root canals [47,48]. Nevertheless, such an approach has been criticized since the areas examined by SEM are considered “too small” compared to the entire space of the root canal system. Moreover, assigning scores to the degree of smear layer removal may be biased by lack of objectivity due to possibly divergent interpretations of each surveyor [49,50]. In order to reduce such possibly negative aspects, we acquired images at different magnifications to permit a more wide-ranging analysis of each specimen in each portion of its root canal system. Furthermore, a proper calibration procedure was used between the examiners before performing images assessment. Such process resulted suitable indeed, showing a Kappa test with a level higher than 85% [51].

Regarding the assessment of sealer penetration inside tubular dentin, confocal scanning microscopy allows the most precise assessment as the specimen’s preparation generates less risk of creating artefacts, as compared to SEM [52,53].

It is essential to highlight that the aim of this in vitro study was to evaluate the efficacy of an innovative silver citrate BioAKT endodontic irrigant on smear layer removal and sealer penetration at different root canal levels (e.g., apical, middle, cervical) after endodontic instrumentation compared to 17% EDTA and 5.25% NaOCl. Consequently, in order to have a “straight” comparison between the tested materials, all specimens received a standardized protocol for irrigation, including treatment with the tested irrigant solutions only at the end of the mechanical root canal instrumentation (10 mL for 1 min).

Given the results of this in vitro study and notwithstanding its limitations, the first hypothesis that the novel root canal irrigant would be able to remove more smear layer removal and achieve better sealer penetration at different root third levels compared to EDTA and NaOCl must be partially accepted. In fact, the use of the novel irrigant could facilitate the removal of smear layer and allowed better sealer penetration at the apical root level after endodontic instrumentation, when compared to 17% EDTA and 5.25% NaOCl. The second hypothesis of the study that the novel root canal irrigant would have a comparable antibacterial activity to the gold-standard treatment such as 5.25% NaOCl against *Enterococcus faecalis* biofilm must be accepted.

## Figures and Tables

**Table 1:** Smear layer removal—Scoring criteria.

Score	Criteria
0	No smear layer, all dentinal tubules open with erosion of tubules.
1	No smear layer, most of the dentinal tubules open.
2	Minimum smear layer; >50% dentinal tubules visible.
3	Moderate smear layer; <50% of dentinal tubules open.
4	Heavy smear layer; outline of dentinal tubules obliterated.

**Table 2:** Sealer penetration—Scoring criteria.

Score	Criteria
0	No penetration of the sealer into the dentinal tubules.
1	Penetration of the sealer into dentinal tubules <50% of the entire perimeter of the canal.
2	Penetration of the sealer into dentinal tubules >50% of the entire perimeter of the canal.
3	Penetration of the sealer into dentinal tubules 100% of the entire perimeter of the canal.
